# ‘Just normal’, ‘calming’ and ‘well looked after’: a qualitative exploration of adolescents’ constructions of active mobility and place in Australia

**DOI:** 10.1093/heapro/daaf155

**Published:** 2025-09-29

**Authors:** Himashini Whitley, Shannon Sahlqvist, Octavia Calder-Dawe, Anna Timperio, Jenny Veitch

**Affiliations:** Deakin University, Institute for Physical Activity and Nutrition (IPAN), School of Exercise and Nutrition Sciences, Djilang, Geelong 3216, Australia; Deakin University, Institute for Physical Activity and Nutrition (IPAN), School of Exercise and Nutrition Sciences, Djilang, Geelong 3216, Australia; Te Herenga Waka, Victoria University of Wellington, School of Health, Te Whanganui-a-Tara, Aotearoa, Wellington 6012, New Zealand; Deakin University, Institute for Physical Activity and Nutrition (IPAN), School of Exercise and Nutrition Sciences, Djilang, Geelong 3216, Australia; Deakin University, Institute for Physical Activity and Nutrition (IPAN), School of Exercise and Nutrition Sciences, Djilang, Geelong 3216, Australia

**Keywords:** youth, active living, adolescent, active transport, health behaviour, children

## Abstract

The active mobility experiences of adolescents intersect with those of younger children and older youth in many ways. However, existing research informing health promotion is limited in its exploration of the distinct features that differentiate adolescent active mobilities and the differences within adolescence. Drawing on interviews and audio-recorded walking tours with 12 adolescents aged between 12 and 15 from urban areas of Australia, this paper examines adolescents’ constructions of active mobility and place in their local areas. We adopted a constructionist epistemological approach and conducted a reflexive thematic analysis of adolescents’ accounts. Our analysis suggests that adolescents construct mobility as an everyday, utilitarian practice, and understand clean, well-maintained places to constitute a ‘good’ neighbourhood. Unlike younger children, our analysis illustrates how urban risk was also constructed as an everyday aspect of mobility that adolescents could navigate with little effort. Mobility as offering connection (both socially and to self) was also a key theme generated by our analysis as a potentially distinct aspect of local active mobilities during the adolescencent years. Based on this, we recommend further critical enquiry into opportunities for enhancing connection in mobility among adolescents from diverse population groups.

Contribution to Health Promotion

- Critically examines how adolescents move around their local areas and how this shapes their lives.
- Highlights how everyday ways of moving around can foster social connection, a sense of self and independence amongst adolescents when these experiences are well-supported.
- Draws attention to age-specific and socio-economic differences in adolescents’ experience of their local areas.
- Suggests that more inclusive, equity-focused health policies could genuinely improve adolescents’ lives in multiple, meaningful ways.

## INTRODUCTION

The transition to adolescence is considered a critical window for public health intervention and research (VicHealth 2015). Within a mobility context, adolescents’ active mobilities in urban areas are explored as distinct yet related health-promoting practices. These practices include independent mobility, active travel and active play. Independent mobility is defined as children’s freedom to move around public space without adult supervision and often increases during the transition to adolescence (Hillman *et al*. 1990). Active travel is defined as walking, cycling or otherwise getting to a destination without the use of motorized vehicles (Pont *et al*. 2009). In Australia, the transition to more independent mobility in adolescence corresponds with an increase in active travel (Australian Bureau of Statistics 2013). In addition to this link, adolescents’ active travel practices, particularly to school, have been the focus of an extensive body of literature on the premise that they present a convenient and inexpensive means of accumulating physical activity (Tudor-Locke *et al*. 2001). Active play refers to unstructured and enjoyable physical activity (Pellegrini and Smith 1998) and also forms an important aspect of adolescents’ urban active mobilities when it occurs in public space. Despite increases in active travel and independent mobility during adolescence, low levels of overall physical activity among adolescents (Hesketh *et al*. 2023) remain a public health concern. The intersecting active mobility practices of active travel, active play and independent mobility have thus been examined and promoted as key sources of physical activity and good health (Larouche 2014, Poitras *et al*. 2016, Condello *et al*. 2017).

Working from a critically constructionist approach that aims to centre adolescents’ own understandings, in this research we adopted the broader concept of ‘active mobility’ to encompass adolescents’ active movements within and between public spaces (i.e. inclusive of active travel, active play in public space and independent mobility). This recognizes the potential incongruence between adolescents’ mobility practices and academic categories (Evans 2008). For example, one study found that adolescent mobilities often involved wandering around in circuitous patterns, partially due to being moved on from intended destinations (Horton *et al*. 2014). It is unclear whether this practice should be understood as active travel, active play, or both. Additionally, none of these constructs may align with how the practice is understood by adolescents (Evans 2008). We acknowledge that concept of ‘active mobility’ remains limited in its capacity to explore adolescents’ own understandings and delimits active from non-active adolescent mobilities.

While extant enquiry into children’s active mobilities has made important contributions towards understanding adolescent movements, research and policy has tended to focus on younger children (Aranda-Balboa *et al*. 2020, Schönbach *et al*. 2020, Smith and Kraftl 2024), particularly those of primary school age (Schönbach *et al*. 2020). In many cases, adolescents (10–19-year-olds) are grouped within the broader categories of ‘children’ (under 18-year-olds), ‘young people’ (10–24-year-olds), or ‘youth’ (15–24-year-olds) with a lack of sensitivity to differences within each category. For example, some reviews and studies present combined findings related to children and adolescents (Pont *et al*. 2009, Lu *et al*. 2014, Jia *et al*. 2021). Neglecting to examine the diverse mobilities of adolescents may lead to health promotion policies that fail to sufficiently meet their needs (Evans 2008).

Research with younger children and adolescents tends focus on limited outdoor play and the transition to independence (Hillman *et al*. 1990, Valentine 1997, Kullman 2010, Alexander *et al*. 2014). Studies suggest that both younger children’s and adolescents’ active school travel may be sensitive to factors such as the distance between home and school (Lu *et al*. 2014, Aranda-Balboa *et al*. 2020) and parental perceptions of traffic safety (Aranda-Balboa *et al*. 2020). Such findings are often understood within the context of parental constructions of risk, whereby parental fears over traffic and strangers (Ikeda *et al*. 2018) are seen to reflect the importance of macro-level influences such as road crossings (Timperio *et al*. 2015) and neighbourhood social cohesion (Lin *et al*. 2017). Parental fears are understood to culminate in the restriction of child and adolescent mobilities, particularly younger children’s independent active mobilities (Hillman *et al*. 1990, Valentine 1997, Forsberg *et al*. 2023). Concerns about parental risk aversion are also reflected in research exploring the restriction of active play (Gill 2007, Sharpe *et al*. 2020).

Although there are shared characteristics in the mobility practices of adolescents and younger children, adolescence may introduce important differences. For example, one review suggested that parental perceptions of social support may be less relevant for adolescents’ active school travel (Aranda-Balboa *et al*. 2020). Another review suggested adolescents may not respond to the same health promotion strategies as younger children (Schönbach *et al*. 2020). The geographies of older youth also expand and vary considerably including wider public transport based mobilities where identity formation and friendships become a feature (Skelton 2013, Smith and Kraftl 2024). Studies including older adolescents suggest that this age group may be constructed as problematic and unruly sometimes resulting in their exclusion from public places despite their independence from adults (Malone and Hasluck 2002, Skelton 2013). This is an aspect of mobility that appears to begin at early adolescence (Malone and Hasluck 2002, Horton *et al*. 2014).

In this study, we focus specifically on the local active mobilities of adolescents in their first 2 years of secondary school. Adolescents who have recently transitioned to secondary school may be a particularly important group to study, given recent increases in their independence and active travel (Australian Bureau of Statistics 2013, Carver *et al*. 2013). We present a reflexive thematic analysis drawing on data collected with 12 Australian adolescents aged 12–15 years old to examine how adolescents’ constructed their local active mobilities and experience of place. We consider both constructions of mobility and place, understanding these to be intermingled: mobility necessarily moves through place, and the experience of place as a destination may also interact with mobility as a journey. Herein, we use the terms ‘adolescents’ to refer to study participants. We acknowledge that this terminology is contested and remains laden with understandings beyond age-based definitions (e.g. adolescence and youth may be associated with a lack of good judgment) (Evans 2008).

## METHODS

This study was informed by a social constructionist position to examine adolescents’ accounts of their local active mobility experiences as constitutive of how they understand mobility and place. We adopted a participatory approach in line with a critical-theory based view that adolescents’ perspectives are lacking in research about their own mobilities (Evans 2008, Riazi *et al*. 2022). This approach aimed to provide participants with greater power over the research process and a variety of methods by which to express their understandings (Shier 2001, Barker and Weller 2003). However, we acknowledge that this analysis remains framed within the adult-dominated academic enquiry into adolescent health and that participatory involvement was limited to data collection. As a group of academics and former practitioners, most of whom have worked from a positivist perspective to examine and encourage active mobility, we also acknowledge our understandings of mobility have shaped the analysis of adolescent experiences.

### Research context

This study was conducted in the inner-city and middle-suburbs of Naarm, Metropolitan Melbourne in Australia, which spans between 40 km to the west and north of the CBD (Central Business District), to over 100 km east of the CBD. The areas selected for this study comprised the more densely populated, older inner-city suburbs of Naarm, Melbourne within 15 km of the CBD, and the middle-suburbs within 15–30 km of the CBD.

### Recruitment

This study draws on data from 12–15-year-old adolescents collected during a follow-up time point in a longitudinal qualitative study. Participants (*n* = 15) were initially recruited at 11–13 years of age when they were in Grades Five or Six at primary school. Recruitment comprised information sessions and/or distribution of study information at two primary schools (*n* = 6), parent-targeted methods including social media posts and community flyers (*n* = 3), and contacting research participants from an existing database who had consented to re-contact (*n* = 6). The original baseline data collection aimed to explore constructions of mobility and place amongst children prior to their penultimate years of primary school and adopted similar data collection methods to the present study. A maximum variability sample including participants from a wide range of backgrounds and diverse active mobility experiences (including those who did not actively move around their neighbourhoods) was initially sought. However, due to difficulty recruiting schools and individuals, all eligible participants who agreed to join the study were included. Detailed information about the original study and recruitment has been published elsewhere (Whitley *et al*. 2025b). Each of these participants was re-contacted approximately 2 years later at approximately the same time of year as their initial participation (September–October 2021, and September–October 2022). Parental consent and adolescent assent to participate in the follow-up study were obtained. Data collection in 2021 coincided with an end to over 18-months of COVID related travel and school restrictions. This included state-wide school and park closures, travel bans and restrictions on social interaction. Overall, 12 adolescents participated in the follow-up.

Ethical approval for this research was granted by a University Ethics Committee and approval to engage with Victorian Government Schools was provided by the Victorian Department of Education and Training.

### Data collection methods

All participants were invited to complete a travel and demographic survey at baseline and follow-up. At both time points, surveys requested basic demographic information (e.g. date of birth, home address and number of siblings) and details about how participants travelled to school and other places. Participants were also asked to identify their gender, nationality and main language(s) spoken at home in the baseline survey. Only 9 of the 12 participants returned a survey at follow-up. The three participants who had not completed the survey were asked to provide this information during interviews, with responses recorded by H.W.

All participants were posted a printed instruction booklet and parents emailed a digital version of the instructions for data collection. Participants were asked to prepare and upload an audio recording of a 10–15-min walking tour around their neighbourhood, and a hand-drawn or digitally produced map of their walk. Participants were asked to act as a tour guide and include details of what they saw, heard, felt and smelt during their tour. We intentionally omitted further instruction on walking tour route selection (i.e. if participants frequented the route) as the purpose was to examine how adolescents talked about active mobility and place (rather than what they talked about).

All participants provided an audio-recorded walking tour. Nine participants recorded their tour without the aid of a parent, including three who recorded their walk in short bursts (commenting at several time points during one walk), four who completed a continuous recording of their entire walk, and two who recorded their tour while at home (i.e. reflecting on a walk). Three participants recorded their tour while being prompted by or in discussion with their mother, including one who provided a video tour recorded by their mother, and one who completed a riding tour while their mother recorded the ride. The video tour contained little talk by the adolescent participant, despite parental prompting.

Eleven of the 12 participants provided an annotated Google map of their tour. Three participants had annotated multiple routes that they used for the same walk (e.g. different ways they could walk home from to the bus stop) or multiple walks or rides they normally performed. Maps were used to aid interview discussions.

Participants were also given the option of uploading drawings, photographs or other information about their local active mobility experiences. Parental consent and adolescent assent included permission to publish a de-identified version of provided materials. The instruction booklet included guidance on appropriate use of photography (e.g. requesting permission before taking photos of other people). Two participants provided photographs and none contained images of people’s faces. All uploads (including audio recordings and annotated maps) were made to a password-protected participant-specific OneDrive folder accessible only to the individual participants’ parent and H.W. The uploaded data were then moved to a secure Deakin University server for research data analysis and storage.

Participants later took part in a 20–30-min semi-structured Zoom interview to provide more detail about the experiences recorded in their walking tour. The audio-recording was used as a starting point to discuss adolescents’ active mobility experiences and were particularly important for shorter recordings or if the adolescent had talked little during a parent prompted tour. If time permitted, discussions also extended to other times adolescents had walked, ridden a bike, or actively moved around their local areas. Interview prompts included requesting details about what they had seen, heard, smelt, and felt during their walking tour, what was different/same from other instances when they had previously walked or actively moved along the same route, and when and how often they used the route. Most interviews were conducted within 2 weeks of recording the walking tour (*n* = 10) however, two were conducted within 3 months due to limited participant availability. All Zoom interviews were conducted by H.W., audio-recorded and transcribed by a transcription service. Prior to analysis and storage, all participants were assigned gendered pseudonyms based on travel and demographic surveys (Hannah, Sally, Kelly, Mel, Nicholas, Nate, David, Ella, Mark, Phil, Tom, and Zoe).

### Data analysis

The analysis presented here is an inductive and reflexive form of thematic analysis whereby the themes represent patterns of shared meaning identified within the data (Braun *et al*. 2019). In this type of thematic analysis, themes are not considered to pre-exist in the data. Instead, themes are interpretive stories that are a product of the analytic process. This process is necessarily subjective. It is therefore expected that different themes could be generated if other analysts considered the same data, or if the same data were analysed through a different lens (Braun *et al*. 2019). Within this context, sample size was defined by practical considerations related to study timelines, reflexive journalling during data collection, and a preliminary review of data by H.W. The preliminary review suggested sufficient depth for the selected analytic approach, and relevance of interview discussions to exploring constructions of active mobility and place (Malterud *et al*. 2016, Braun and Clarke 2019).

To conduct this analysis, H.W. reviewed each interview transcript (*n* = 12), audio-recorded walking tour transcript (*n* = 12), and photograph (*n* = 14). Initial familiarization with the data included broadly organizing data into participant’s constructed understandings of place or of mobility, as well as other constructions that did not fall within these two categories. Data were then thematically coded into different constructions using NVivo 14.24.0. H.W. initially coded semantically (explicit meaning) and latently (implicit) to generate a range of codes. Codes were grouped, ungrouped and re-grouped through writing and repeated reading of coded sections to identify patterns of shared meaning that formed the themes presented below (Braun *et al*. 2019). Photographs were similarly coded based on descriptions provided during interviews. H.W. led this analysis with input of from O.C. This collaborative stage focused on latent meanings in line with the research aims to explore adolescents’ constructed understandings of mobility and place. H.W. also maintained a reflexive journal throughout data collection and analysis. This included reflecting on theme development and their interaction with H.W.’s own understandings of adolescence, active mobility and urban environments.

### About the participants

All participants were in Years Seven or Eight and aged between 12 and 15 years. While two participants had relocated during the transition to secondary school, all resided in areas within the top three quintiles for relative socio-economic advantage and disadvantage in Victoria at both time points (Australian Bureau of Statistics 2018). Six had identified or were identified by their mothers as boys at baseline, and six had identified or were identified by their mothers as girls.

Based on the travel and demographic surveys completed by participants, a summary of participant demographic and travel characteristics are provided in Table 1. Additionally, all participants actively commuted to other destinations on at least some days of the week.

**Table 1.** Participant demographic and travel characteristics.

Participant pseudonym	Age	Walked, cycled or used an active mode for all/part of the journey to school^a^	Used public transport for part of the school journey^b^	Usual accompaniment on the school journey	Resided in inner-city (I), or middle-suburbs (S), and an area of high socio-economic advantage (H) or area of low socio-economic advantage (L)
Hannah	14	Every day	Yes	On their own	IH
Sally	15	Every day	No	On their own, or with a parent/other adult	IH
Kelly	14	Every day	No	With a friend/other child	IH
Ella	15	**Never/rarely**	Yes	On their own	SH
David	13	**Never/rarely**	Yes	With a parent/other adult	IH
Mark	14	**Never/rarely**	Yes	On their own	SH
Phil	13	Every day	Yes	With a friend/other child	SH
Mel	13	Every day	Yes	With a friend/other child	IH
Nate	13	Some days	Yes	With a parent/other adult	IH
Nicholas	14	**Never/rarely**	No	With a parent/other adult	IH
Tom	12	**Never/rarely**	Yes	On their own	SH
Zoe	13	Every day	Yes	On their own, or with a friend/other child	SH

^a^Never/rarely’ denoted in bold text—While five participants (Ella, David, Mark, Nicholas, and Tom) reported that they never/rarely actively travelled to secondary school, only Nicholas was driven to school. Interviews revealed that Ella, David, Mark, and Tom walked to and from the bus stop to get to school on a regular basis.

^b^Based on discussions with participants during interviews.

## RESULTS

This section outlines three key themes that were produced during the analysis: (i) our place as ‘good’, (ii) everydayness of mobility, and (iii) mobility as connection.

### Our place as ‘good’

Many participants constructed their neighbourhoods as ‘nice’ and ‘good’ places based on understandings of cleanliness, care, amenity and friendliness. Participants often drew attention to specific elements of their local area, constructing these amenities as hallmarks of a ‘good’ neighbourhood. For example, references to care and maintenance, such as ‘beautiful nature strips’ and ‘houses that are really well looked after, and their gardens are really nice’, depicted the presentation of homes and gardens as evidence that participants’ neighbourhoods were exemplary. Such descriptions went beyond explaining what participants saw, heard, smelt or felt (as requested in audio-recorded walking tour instructions and interviews) and applied value judgements to elements of the neighbourhood. As shown by the following phrases, adjectives such as ‘nice’ and ‘many’ constructed specific aspects of place as favourable and valuable:

nice space [to have parties] (Mel).

bike-friendly [infrastructure] (Zoe).

many shops and cafes nearby (David).

Kelly further explained how she felt ‘very lucky’ and ‘grateful’ to live in a neighbourhood with these features.

**Figure 1.**
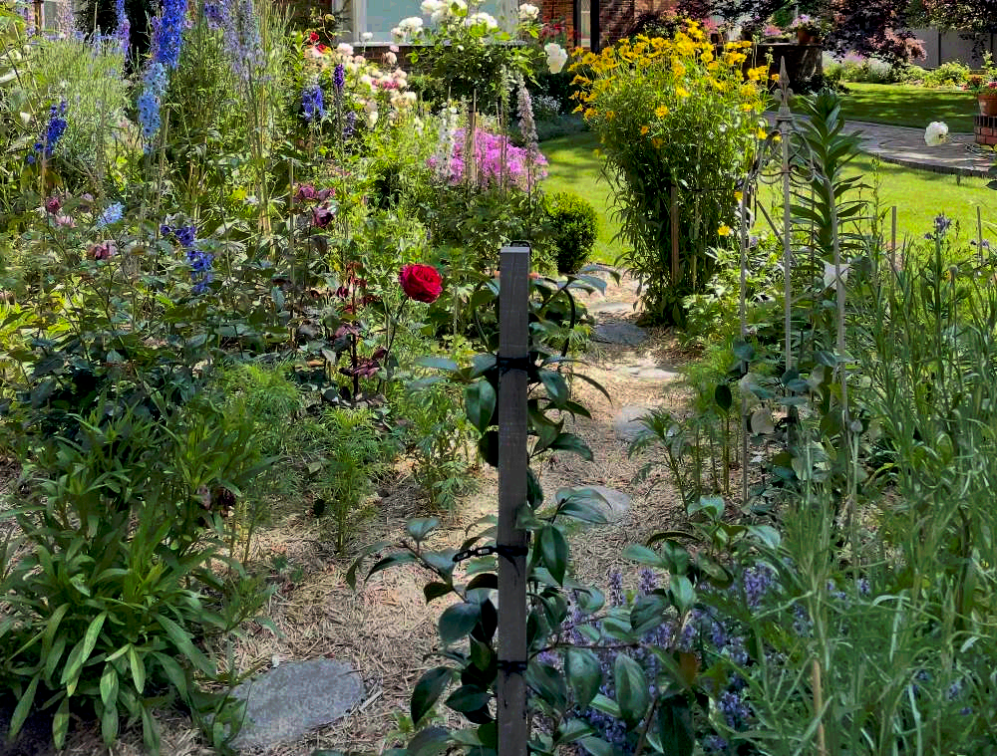
Photograph by Sally of ‘the pretty cottage gardens’.

Natural amenities such as ‘trees and grasses for sniffing for dogs’, ‘trees’ that ‘give us nice shelter’, and ‘beautiful flowers and plants […] [that] makes the area much nicer, and fresh’ were also valued. Within this context, Sally supplied the following image (Fig. 1) of a front yard to illustrate how there were ‘a few gardens’ that were noteworthy and ‘really cool’ on her walk to school.

### Everydayness of mobility

Mobility was most often talked about as everyday, mundane, forgettable, ‘usual’, ‘regular’, and ‘just normal’. Phrases such as ‘pretty much the same every time’, ‘nothing remarkable’ and ‘not a very interesting walk’ that constructed mobility as a mundane and repetitive activity were common across all participant accounts. In part, this reflected mobility being understood as a utilitarian pursuit that primarily got participants from A to B. For example, David described his journey in a list format saying:

[…] And then left. Went through the park for a while. […] Went down this road next to my tennis. And then [we] were in the carpark. (David)

The destination was sometimes constructed as a reward where participants felt they had completed the mundaneness of mobility. For example, Zoe explained how this was the case when walking home from the bus stop after school:

Because once I get through [name of street] […] just like, that’s the house, I can see it, I’m going home [laughs]. (Zoe)

When questioned about any ‘stories’ or ‘memories’ they had, participant descriptions were often suffixed and discounted with ‘that’s about it’ and ‘just’ something that they navigated with little fuss. For example, Mark explained how he ‘just’ took himself on a 13 km walk (one-way) because he was ‘bored’ during lockdown. Others like Kelly, explained how they could not think of anything striking about their walks or rides due to the repetitive nature of mobility:

[…] because I do it [stop at the Seven-Eleven on the way to school] quite a bit, so it’s like, kind of an everyday [walk]. (Kelly)

While adolescents noted how their mobility experiences had changed considerably since primary school (e.g. public transport use, walking for longer distances, travelling with different companions and mobile phones), these changes were often constructed as part and parcel of the everyday experience of mobility. As described by Mel, ‘I have to take the train and the bus and do a bit of walking as well to get to [secondary] school […] But that’s kind of it, yeah’. In the following excerpt from his walking tour, Phil also describes a typical day moving to and from school like a procedure, and merely a way to move from one place to another. The bus trip is constructed as fitting into this everydayness of mobility.

Around seven-forty, seven-fifty, my Mum […] offered to drive me to the bus stop […] I got there, caught the [bus number], down to the closest bus stop to [Name of secondary school] Secondary. Two bus stops after [Name of local train station]. Then I had my regular school day, took a bus from school to basketball with a friend. (Phil)

**Figure 2.**
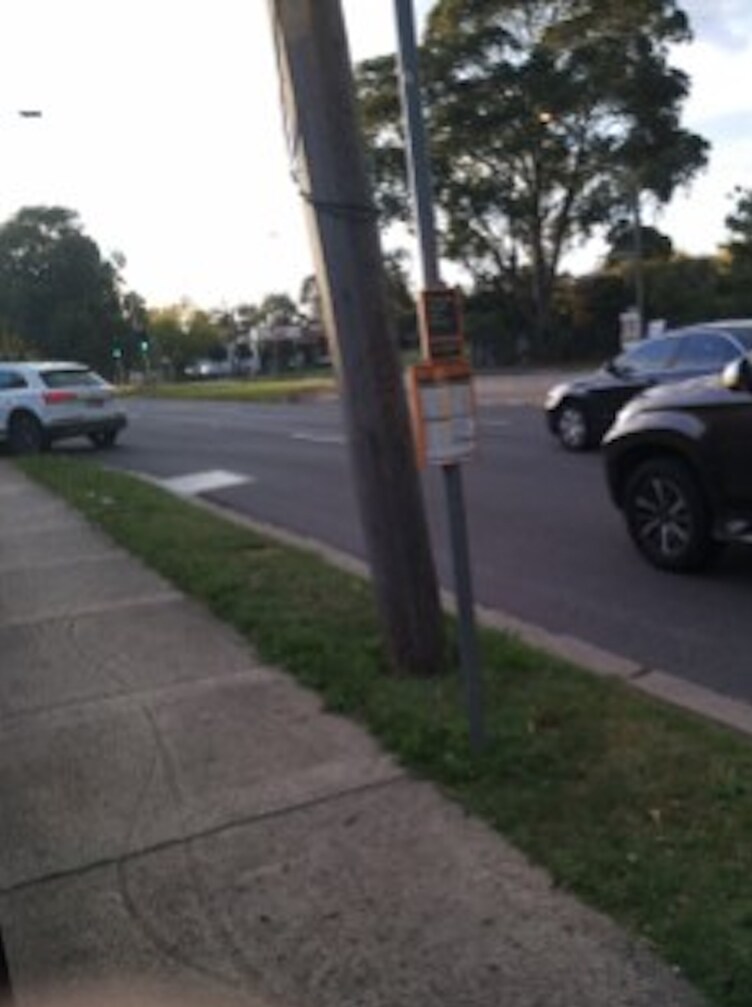
Photograph by Ella of her local bus stop.

Ella also provided two photographs of a bus stop she uses to get to school (including the photograph shown in Fig. 2 below). However, when asked about the images she dismissed their importance saying that she had ‘just taken two photos’.

Similarly, on the handful of occasions where adolescents mentioned their mobile phones, they appeared to be seamlessly integrated into conversations as a natural extension of mobility. Phrases like ‘I’ll text her when I come down to the main street, so she knows […] when to come and pick me up’ and the following excerpt from Zoe’s interview constructed the phone as supportive of the everyday processes of mobility. However, mobility when aided by technology was constructed as obvious and no big deal. As noted by Zoe, she ‘just’ waits because she can ‘track the bus’ using her mobile phone.

Zoe: […] it’s [referring to the bus] just right at the very corner.

HW: […] And what happens when you’re waiting [for the bus]?

Zoe: I just wait [laughs]. So, I can track the bus on my phone so, yeah.

Similar incidental references to the mobile phone and its accepted role in mobility were common across many participant accounts.

While COVID related changes were often constructed as different from the norm, these differences were rarely constructed as either challenging or enabling. Instead, participants presented these COVID time variations as a mere change in the organization of time, in the same way that changes since primary school were ‘not a big deal’. For example, in the following quote, Hannah constructs mobility as being different during lockdown and explains how the presence of ‘sketchy’ people around the neighbourhood during COVID restriction periods ‘changed’ how she moved around post restrictions. These changes were however, dismissed as being of little importance with phrases like, ‘that’s about it’. In this way, COVID time mobilities also reflected constructions of mobility as everyday.

HW: Okay, […] about lockdowns, but what changed about how you moved around your neighbourhood more broadly […]?

Hannah: Deﬁnitely going on more bike rides and just being out more, I think. Because also for a bit there it got a bit, I kind of got a bit, there were some people that were a bit sketchy, so it kind of changed the way I walked around, went on different paths and stuff. But that’s probably about it.

Navigating urban risks also appeared to be constructed as just a part of urban mobility. Phrases such as, ‘there’s a lot of cars’, ‘a lot of traffic’, ‘the roads I cross are very wide’ and ‘we try to avoid the main roads’ constructed car drivers and local traffic as a risk that participants were both aware of and capable of managing. However, these urban risks were understood to be an everyday feature of mobility that ‘feel fine’ and are not necessarily important enough to ‘remember’. As shown in the following quote, participants constructed the repetitiveness of a journey as extending to the urban risks that participants were comfortable with navigating, often on a daily basis.

[…] I think it [footpath next to a busy road] feels fine because I’m used to it. (Nicholas)

When reflecting on how mobilities had changed since primary school, participants often related risk management to their own age-related ability to ‘read’ the street environment. For example, comments like ‘you have to have a lot more responsibilities and skills as well [in secondary school]’ subtly suggested that adolescents understood their own capacity in relation to broader constructions of child development. In the following quote, David links a more flexible approach to mobility to his current age and developmental stage. He constructs himself as less knowledgeable of the neighbourhood when at primary school and more comfortable with navigating the local environment at secondary school.

I ride more. I used to never even go that much. Like, at the most I’ll go down to the bottom of my street by myself and now I go way further. I also know where I am. (David)

Like David, many participants talked about their mobilities as relatively flexible and often included maps showing different ways they might get to the same location. Participants explained that route choice depended on who they were with and where they wanted to go to on that day.

### Mobility as connection

All participants constructed mobility as offering connection and joy. This was often depicted as contingent upon who was around, what was happening, where participants were (or were going to) and when experiences occurred. Connection included the social form with ‘friends’, and ‘other people’, connecting with self on long walks or rides on their own, and connecting with place.

Many participant descriptions specifically linked mobility with social connection. In the following excerpt, Hannah constructs active mobility as primarily health related, but able to provide connection and joy when she was with friends. Her quote illustrates how connection appeared to shift mobility from being an everyday, utilitarian activity ‘for exercise’, to something that was also ‘enjoyable’, ‘fun’ and offered a sense of fulfilment or purpose.

Hannah: When I’m by myself, then I’ll ride faster. But when I’m with friends, usually it will be like a slower ride.

[…]

HW: Okay, and how do you feel riding on your own on that trail, and coming back?

Hannah: Usually pretty good because there’s always people there.

HW: And is the feeling different when you ride with a friend?

Hannah: Yeah, because I talk to them, so it’s different. When I ride by myself it’s more just for exercise, but when I ride with my friend it’s for fun as well.

Some adolescents appeared to value social connection as an alternative to what was sometimes described as an ‘embarrassing’ or ‘lonely’ feeling of being ‘one kid, just random, from a school’ walking on their own. As shown in the following quotes, adolescents constructed a sense of discomfort in doing mobility on their own, understanding social connection to support a more enjoyable and less awkward experience.

I kind of wish I had a friend to walk with though. (Sally)

It is very lonely walking by yourself, even though it is a quick walk. (Kelly)

Participants also constructed mobility as slow, calming, relaxing or providing connection to self at secondary school age. Phrases such as ‘it was more peaceful’, ‘trying to focus on more thoughts in my head, the different things around me’, and ‘it can be calming’ constructed mobility as offering a sense of individual connection (to self). Several participants linked feelings of being ‘relaxed and calm’ with a lack of traffic, understanding this to be ‘peaceful’. Some participants related this specifically to the lower volume of cars during COVID restriction periods. For others, this involved finding quieter streets in preference to main roads. Many also reflected on new ways of moving around during COVID restriction periods as providing connection. For example, Mark explained how a long walk, spurred by having ‘nothing else to do’ during COVID, provided him with a sense of relaxation:

I’m bored, so I just walk there. It’s also relaxing. (Mark)

Another aspect of connection was reflected in participants’ constructions of mobility or place as engaging and interesting when place was interpreted/imagined in other ways. This was both in terms of the places they were moving through and the places that were the destination of their journeys. The ‘downhill bits’ were ‘fun’ on a bike, it was ‘fun’ going to the breakwater ‘to climb on the rock and stuff’, and riding ‘very fast’ when the ‘sun is out’ made you feel ‘happy’. The following excerpt shows how David constructed the speed humps on a suburban street as offering a valued experience when interpreted as a bike jump. The sensation of speeding and potentially getting ‘ahead of the cars’ was highlighted as a noteworthy aspect of his ride. While his reference to not ‘quite’ flying ahead subtly constructed his awareness of the need to manage risk, the appropriation of place appeared to offer David an alternative embodied experience to the everydayness of his journey.

Well, because there’s speed bumps, the cars slow down for speed bumps, but I speed as fast as I can at the speed bumps to try and jump over them. So, I usually don’t quite fly ahead of the cars […] (David)

In the below story, Mel described how she and her friends had ‘discovered’ and appropriated a local tree. She constructs this place as being of value and offering connection as a place to ‘meet at’ and watch other people secretively with her friends. Her reference to ‘no one really pays that much attention’ constructs this location as somewhat hidden and known only to whoever is in the tree. The reimagined tree appears to provide a place that is somewhat undesignated, unmonitored and fulfilling.

[…] During lockdown me and my friends discovered this tree […] It’s kind of nice ‘cause like it’s one of those trees that, when you’re up there, you’re kind of between the leaves and you can see out, but people below can’t really see in. […] if you were really looking and you were trying hard to look and you like knew where to look then you would be able to see us, but no one really pays that much attention. (Mel)

**Figure 3.**
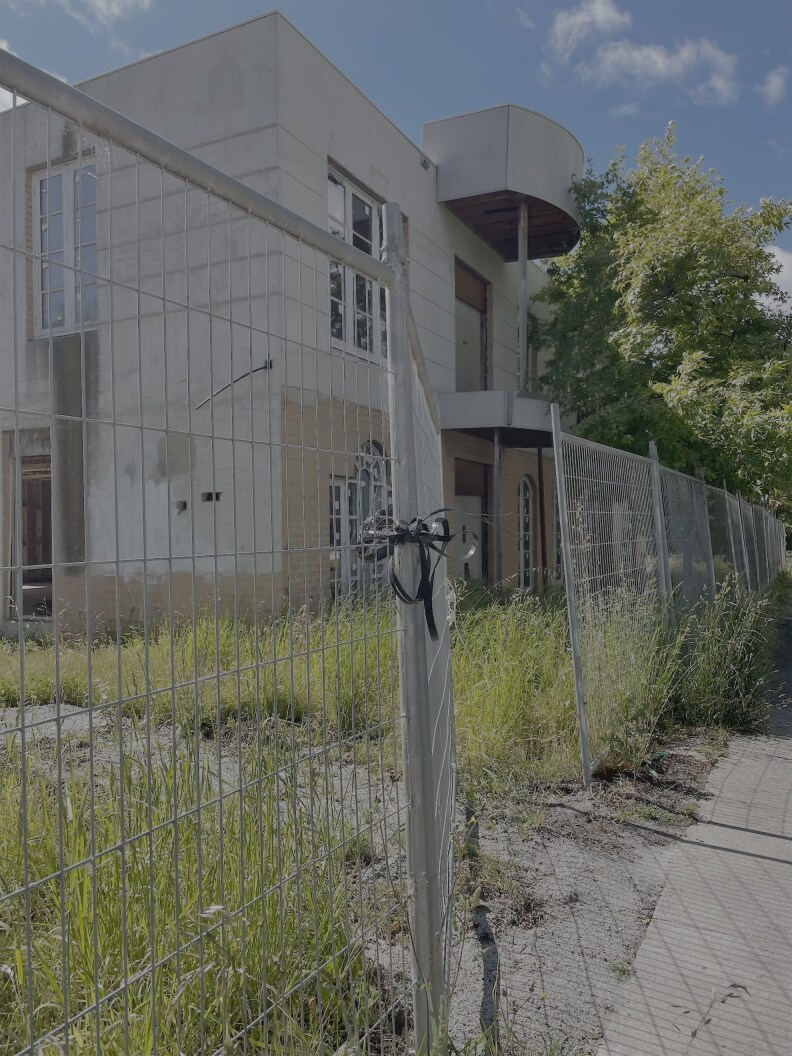
Photograph by Sally of ‘the abandoned building site’.

Likewise, Sally provided the following image (Fig. 3) constructing the interaction with an abandoned house as engaging when imagined as a mystery:

It’s kind of like cool to see the inside, walking past, through the fencing. I just like looking at it. It’s really kind of creepy, but cool at the same time. (Sally, describing the below image)

## DISCUSSION

Our analysis suggests that constructions of mobility and place among secondary-school-aged adolescents reflect dominant understandings of mobility and place. For example, mobility has long been conceptualized as a means to get from A to B (Sheller and Urry 2006). The construction of mobility as an everyday, banal practice has also been observed across studies examining both younger children’s and adolescents’ movements (Horton *et al*. 2014, Whitley *et al*. 2025b). Similarly, the relationship between connection and mobility (particularly social connection) (Middleton 2010, Skelton 2013), and the construction of well-kept and maintained places as signs of a good neighbourhood have been previously documented (Witten *et al*. 2017, Tobin *et al*. 2022).

Understandings of mobility and place seem to shift between primary and secondary school. Compared to when these adolescents were in primary school (Whitley *et al*. 2025b), participants constructed urban risk less as a constant concern and more as a routine aspect of navigating their neighbourhoods. A study with parents of these same adolescents also traced how parents adopted structured approaches to educate and prepare children for greater independence by secondary school (Whitley *et al*. 2025a). The everydayness of managing urban risk amongst adolescents may thus suggest that parents’ efforts enabled children to feel confident in navigating urban risk by early adolescence. However, as noted in a variety of parenting studies, it may not be possible for all parents to enact such structured practices (Smyth and Craig 2017, Whitley *et al*. 2025a). Both parents and adolescents also drew on dominant constructions of child development to justify these changes as ‘normal’ (i.e. the secondary-school-aged adolescents were more capable). This may suggest that commonly accepted developmental understandings support and explain Australian children’s increase in independence by secondary school age. However, we note that many adolescents in our study moved independently between some locations, and understood the management of urban risk to require greater vigilance when they were in primary school (Whitley *et al*. 2025b). Public health promotion activities may benefit from further investigating the link between dominant developmental trajectories and independent mobility to better understand the potential to shift the age at which children’s independence increases.

A change in participants’ emphasis on connection between primary and secondary school was also noticeable. The desire for social connection and feelings of awkwardness when moving around alone were more prominent following the transition to high school. Furthermore, at primary school the link between mobility and connection was apparent only amongst girls whereas, in the present analysis, connection was a feature of discussions with both adolescent boys and girls. This may suggest that the transition to secondary school marks a period where the link between mobility and connection becomes more important. This is in line with studies of older adolescents and youth where friendships, social encounters and a sense of self appear to be significant in both shaping and being shaped by mobility (Skelton 2013, Smith and Kraftl 2024). Our analysis suggests that supporting active mobility amongst this age group may require a better understanding of how mobility and place can foster connections with others, the environment and self.

Participants in our study rarely constructed connection or their mobilities as being controlled or impeded, even within the constraints of COVID restriction periods. This stands in contrast to research inferring that adolescent mobilities are often regulated to the detriment of their freedom to explore connections in mobility (Skelton 2013, O’Keeffe and Jenkins 2022). For example, studies have shown how adolescents are sometimes moved on by adults from places due to concerns over loitering (Malone and Hasluck 2002, Horton *et al*. 2014), which may result in their withdrawal from public space in favour of spending time at home (Malone and Hasluck 2002). Other research has drawn attention to how health-promoting mobility policies may limit the exploratory nature of adolescent and youth mobilities by constructing certain forms of mobility as illegitimate (e.g. street skateboarding in found places) (O’Keeffe and Jenkins 2022). This control and regulation of mobility has sometimes been linked to disadvantaged neighbourhoods (Malone and Hasluck 2002, Horton *et al*. 2014). However, participants in our study were drawn from mid-high socio-economic areas, a group shown to have higher rates of physical activity and active travel compared to those from disadvantaged areas (Katzmarzyk *et al*. 2016, Smith *et al*. 2017). Participants in our study also had a high level of independence and active travel to school. Six participants (50%) used active modes to get to secondary school every day compared to 31% of secondary school students nationally, and eleven (90%) used active modes on at least somedays of the week compared to 52% of secondary school students in Australia (Active Healthy Kids Australia 2022). Although our sample was small, our analysis suggests that adolescents from mid-high socio-economic backgrounds may be well supported in actively moving around their local areas. This aligns with research suggesting that public health policy may better reflect the understandings of those from mid-high socio-economic backgrounds (Wiltshire *et al*. 2019, Whitley *et al*. 2025a). Our analysis further suggests that being supported in their active mobilities enabled participants from our study to explore connections through mobility. Future research should critically examine how and to what extent connection in mobility is fostered amongst adolescents across a range of population groups.

Participants’ emphasis on connection and their experiences of mobility may also relate to COVID with most participant accounts reflecting on mobility experiences during COVID restriction periods. A separate study involving these adolescents’ parents suggested that some parents encouraged more spatially extensive and flexible independent adolescent mobilities during COVID restriction periods. This related to changes in what was considered risky: concerns over physical inactivity, and high screen and social media use became more prominent during restriction periods compared to pre-COVID parental concerns over urban risk (Whitley *et al*. 2025a). It is therefore possible that the unique circumstances of COVID both facilitated and heightened the importance of connection in mobility for adolescents in our study. This aligns with findings from a New Zealand study with 16–24-year-old participants where active travel became a valued activity during COVID restriction periods, partly as a means for social connection (Trask *et al*. 2023). Further enquiry is required to examine these links in more detail in the absence of COVID restrictions. While online environments may play an important role in fostering connection (Armstrong-Carter and Telzer 2021), exploring connection in mobility could also help address problematic social media use among adolescents (Montag *et al*. 2024).

### Limitations

Our analysis is limited by a sample of participants residing in middle and upper socio-economic urban areas in Australia for whom the main language was English. Further enquiry is required to examine how adolescents from different population groups, such as those from lower socio-economic backgrounds or rural areas, may construct mobility and place. This may be particularly important given research suggesting that adolescents from disadvantaged neighbourhoods often experience mobility, place and urban risk in distinct ways (Malone and Hasluck 2002, Pain 2006, Horton *et al*. 2014). In line with a constructionist approach, the themes presented in this paper are further considered to be a product of the analysis process and thus cannot be considered exhaustive. Instead, the coherence, clarity and quality of the themes presented in this paper reflect the suitability of the depth and volume of collected data to draw meaningful insights from the selected analytic approach (Malterud *et al*. 2016, Braun and Clarke 2019).

## CONCLUSION

Our analysis suggests that adolescents residing in upper and middle socio-economic urban areas understand mobility to be a mundane, everyday, utilitarian aspect of their lives. However, our analysis highlights some key differences during adolescence. Notably, the link between mobility and social connection—as well as mobility’s connection to a sense of self and affinity with nature—appear to grow in importance. The value of connection during adolescence may relate to a reconfiguration of social interactions during COVID restriction periods in Naarm, Melbourne, Australia. We suggest that policymakers should remain cognisant of how policy and practice may facilitate and hinder adolescents’ opportunities to explore connections in mobility, with future research to critically examine health promotion efforts in this regard. Public health promotion could also benefit from exploring a diverse range of adolescent age groups and their understandings of place and mobility to identify features specific to this demographic. In particular, we suggest a focus on the experiences of adolescents from disadvantaged areas where existing health promotion activities may lack resonance.

## Data Availability

Not applicable.
